# Chronic Osteomyelitis of the Femur: A Rare Manifestation of Melioidosis

**DOI:** 10.7759/cureus.84775

**Published:** 2025-05-25

**Authors:** Abhijathya Chintha, Pavan Gowda M Y, Sharada V Kutty, Mohd Asif, Padmaja Nama

**Affiliations:** 1 General Medicine, All India Institute of Medical Sciences, Mangalagiri, IND; 2 Microbiology, All India Institute of Medical Sciences, Mangalagiri, IND

**Keywords:** burkholderia pseudomallei, clinical infectious medicine, hyponatremia, melioidosis, osteomyelitis

## Abstract

Melioidosis is an infectious disease caused by *Burkholderia pseudomallei*. While pulmonary and cutaneous manifestations are common, osteomyelitis is a relatively rare presentation. This case report describes a middle-aged man who presented with fever, fluctuating sensorium, and right knee pain for five months.

Blood cultures grew *B. pseudomallei*, confirming the diagnosis of melioidosis. Imaging revealed osteomyelitis of the right femur as the primary clinical manifestation. The patient's altered sensorium was attributed to severe hyponatremia, a complication often associated with poor clinical outcomes in melioidosis. He was managed with culture-guided intravenous antibiotic therapy for two weeks, followed by oral eradication therapy for three months.

## Introduction

Melioidosis is an infectious disease caused by *Burkholderia pseudomallei, *a Gram-negative, facultative intracellular bacterium and widely distributed environmental saprophyte. Transmission occurs through percutaneous inoculation, inhalation, or ingestion after exposure to contaminated soil or water. The incubation period ranges from 1 to 21 days [1-3]. Melioidosis is a significant public health concern in tropical regions, especially in Southeast Asia and northern Australia. A 2016 global statistical model estimated approximately 165,000 human cases annually, with around 89,000 (54%) resulting in death [4].

In India, melioidosis remains underreported. This may be attributed to limited awareness and restricted access to diagnostic facilities. Since 1991, 583 cases have been documented, mainly in Karnataka and Tamil Nadu [5-11].

Melioidosis most commonly presents as pneumonia; however, it can affect multiple organ systems. Cutaneous manifestations include skin ulcers and soft tissue abscesses, while genitourinary involvement may lead to acute pyelonephritis or prostatic abscesses. Neurological complications, though rare, can include encephalomyelitis, meningitis, cerebral abscess, myelitis, and epidural abscess. Melioidosis, though uncommon, can manifest as periorbital cellulitis or eyelid abscess in the eyes and as septic arthritis or osteomyelitis in the musculoskeletal system [12,13]. 

## Case presentation

A man in his early 40s, from Southern India, presented to the emergency department with fever and right knee pain for five months. He worked as a multipurpose health worker and was a reformed alcohol user. He was a known type 2 diabetic with poor control (HbA1c of 8.6 and retinopathy) on insulin for the last five years.

The patient reported experiencing intermittent fever associated with chills and rigors for five months. At approximately the same time, he began experiencing right knee pain, rated 7/10 (Wong-Baker FACES Pain Rating Scale) in intensity. The pain worsened with movement and improved with rest. Due to the severity of the pain, the patient found it difficult to perform activities of daily living and had impaired sleep quality. 

The patient recalls being exposed to heavy rains and stagnant flood water the week before the onset of symptoms.

Due to persistent symptoms of fever and right knee pain, he was admitted to a tertiary center, where his complete blood picture revealed normocytic normochromic anemia, normal leukocyte count, and thrombocytopenia. He was evaluated for leptospirosis, dengue, scrub typhus, and malaria, which were all negative.

**Figure 1:**
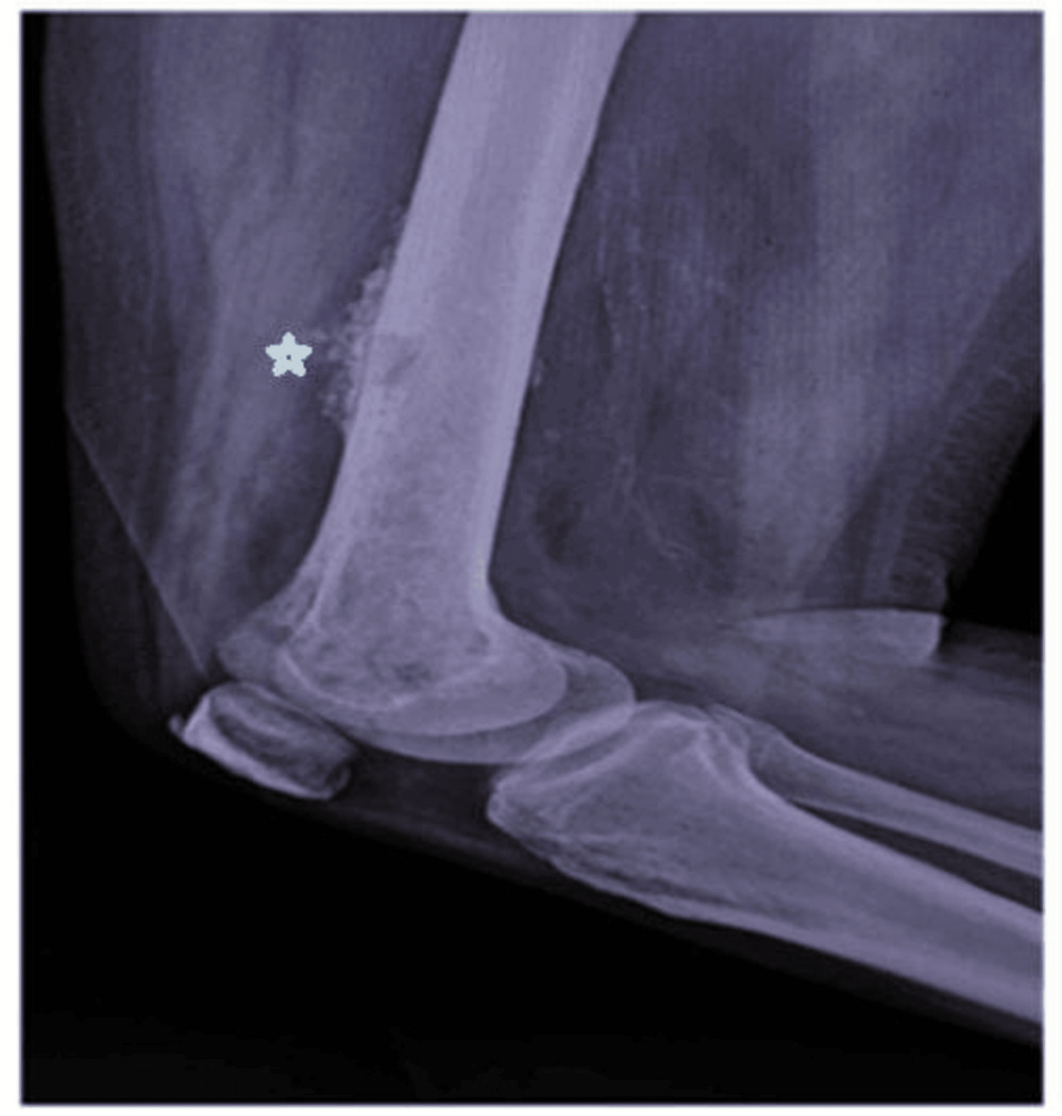
X-ray of the right femur (lateral view) showing cortical irregularity and periosteal thickening of the distal femoral shaft (asterisk) with adjacent soft tissue swelling, suggestive of osteomyelitis. There was no contiguous involvement of leg bones

An X-ray of the right femur showed features suggestive of osteomyelitis, as shown in Figure 1.

Ultrasonography of the right thigh showed a unilocular, thick-walled abscess measuring 8 × 7 cm located on the lateral aspect of the thigh, with associated periosteal irregularity of the adjacent femoral bone, suggestive of osteomyelitis of the distal femur.

**Figure 2:**
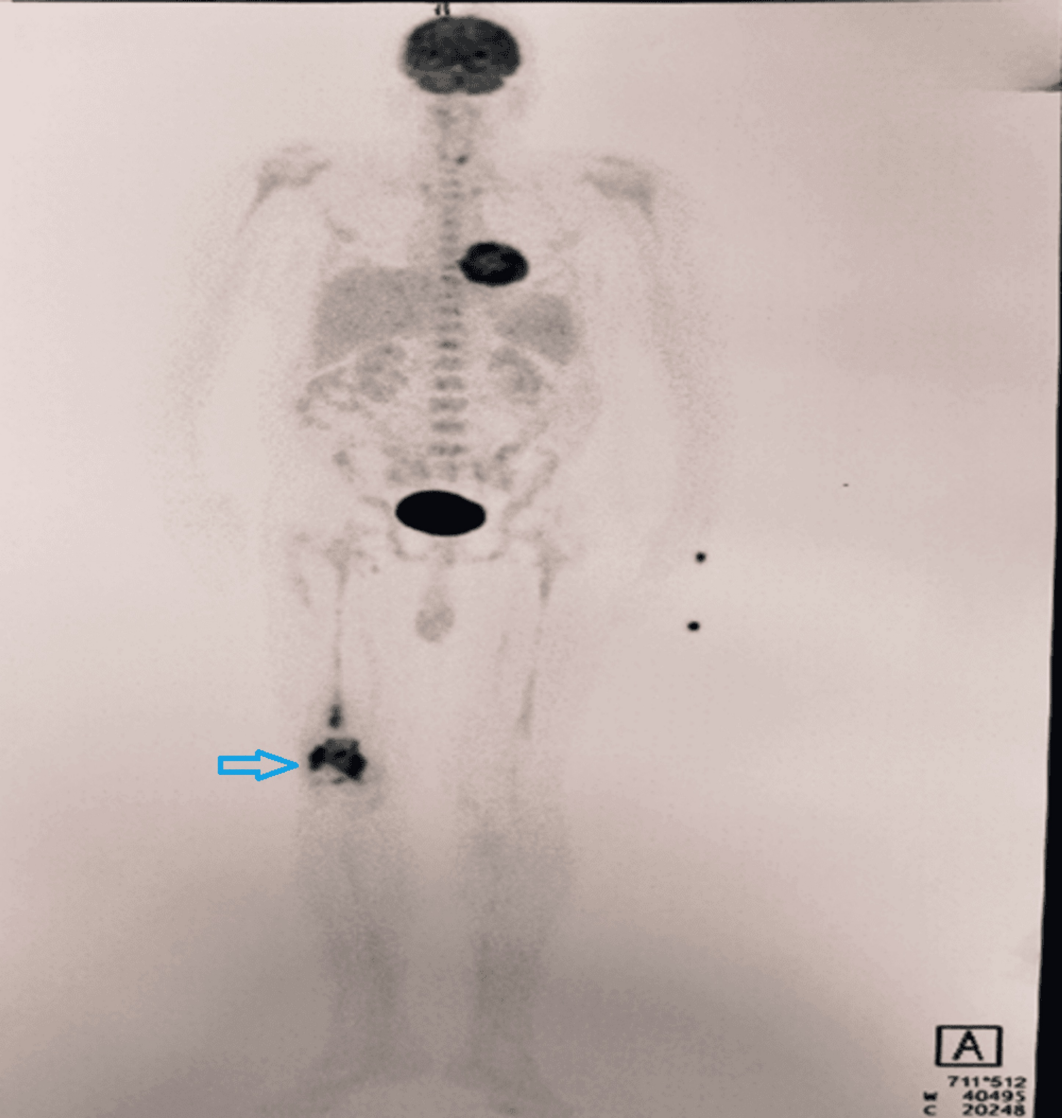
PET-CT maximum-intensity projection (MIP) showing metabolically active lytic-sclerotic changes with soft tissue component seen in distal end/metaphysis of the right femur (arrow), with no evidence of dissemination PET-CT: positron emission tomography-computed tomography.

A PET scan was done to assess for disease dissemination. It revealed metabolically active lytic-sclerotic changes with an associated soft tissue component in the distal right femur, as shown in Figure 2.

He was diagnosed with right knee osteomyelitis and right thigh abscess, for which he underwent surgical debridement with complete removal of purulent material. As per the available medical records, the tissue culture grew methicillin-resistant *Staphylococcus aureus* (MRSA), which was sensitive to linezolid, moxifloxacin, doxycycline, gentamicin, levofloxacin, piperacillin, and tazobactam. He was treated with parenteral linezolid and piperacillin-tazobactam based on culture sensitivity for four weeks. As per history, the patient had a resolution of fever and reduced knee pain. The lab parameters improved, and he was discharged on oral antibiotics (records of which were unavailable).

However, 72 hours after discharge, he presented to our center because of reappearing fever, worsening pain in the right knee and thigh, and confusion.

On examination, he was febrile with an axillary temperature of 101°F, pulse rate of 122 bpm, blood pressure of 112/70 mmHg, respiratory rate of 19/min, and oxygen saturation of 97% on room air. The patient was conscious but confused; his Glasgow Coma Scale (GCS) score was E4V4M6 (14/15). Cardiovascular and respiratory examinations revealed no significant abnormalities. Local examination revealed swelling of the lower end of the right femur with mild tenderness. A previous debridement wound was present with a healthy scar and no pus discharge. Movement of the right knee joint was painful, with mild limitation in the range of movement.

Initial investigations, including blood cultures, were sent. His chest X-ray was normal. He was started on empirical antibiotic therapy with meropenem 6 g/day and teicoplanin 400 mg/day.

His hemogram and basic metabolic panel are shown in Table 1.

**Table 1:** Laboratory blood investigations ALT: alanine transferase, AST: aspartate transferase, ALP: alkaline phosphatase, GGT: gamma-glutamyl transferase, HbA1c: glycated hemoglobin.

Lab investigations	Results	Reference value
Hemoglobin	7.5 g/dL	13-17 g/dL
Leukocyte count	6,500/uL	4,000-10,000/ µL
Neutrophils	91%	40%-80%
Lymphocytes	5%	20%-40%
Platelet count	150,000/uL	150,000-410,000/µL
Serum creatinine	1.1 mg/dL	0.6-1.1 mg/dL
Blood urea	40 mg/dL	12.8-42.8 mg/dL
Sodium	120 mEq/L	136-145 mEq/L
Potassium	3.8 mEq/L	3.5-5.1 mEq/L
Chloride	90 mEq/L	98-107 mEq/L
HbA1c	8.6%	<6.5%
Total bilirubin	0.8 mg/dL	0.2-1.1 mg/dL
ALT	44 U/L	18-78 U/L
AST	100 U/L	18-54 U/L
ALP	108 U/L	50-116 U/L
GGT	45 U/L	13-109 U/L
Calcium	7.4 mg/dL	9.1-10.4 mg/dL

The patient had severe euvolemic hyponatremia. In this case, two important considerations concerning the cause of hyponatremia were evaluated: linezolid-induced hyponatremia versus disease-associated hyponatremia. The hyponatremia was managed with an infusion of hypertonic saline over the next 48 hours, resulting in significant improvement in sensorium. No recurrence of hyponatremia was observed thereafter.

An MRI of the brain was performed and was found to be normal. Fundoscopy and 2D echo were negative for signs of infective endocarditis. A baseline Widal was performed and found to be negative.

**Figure 3:**
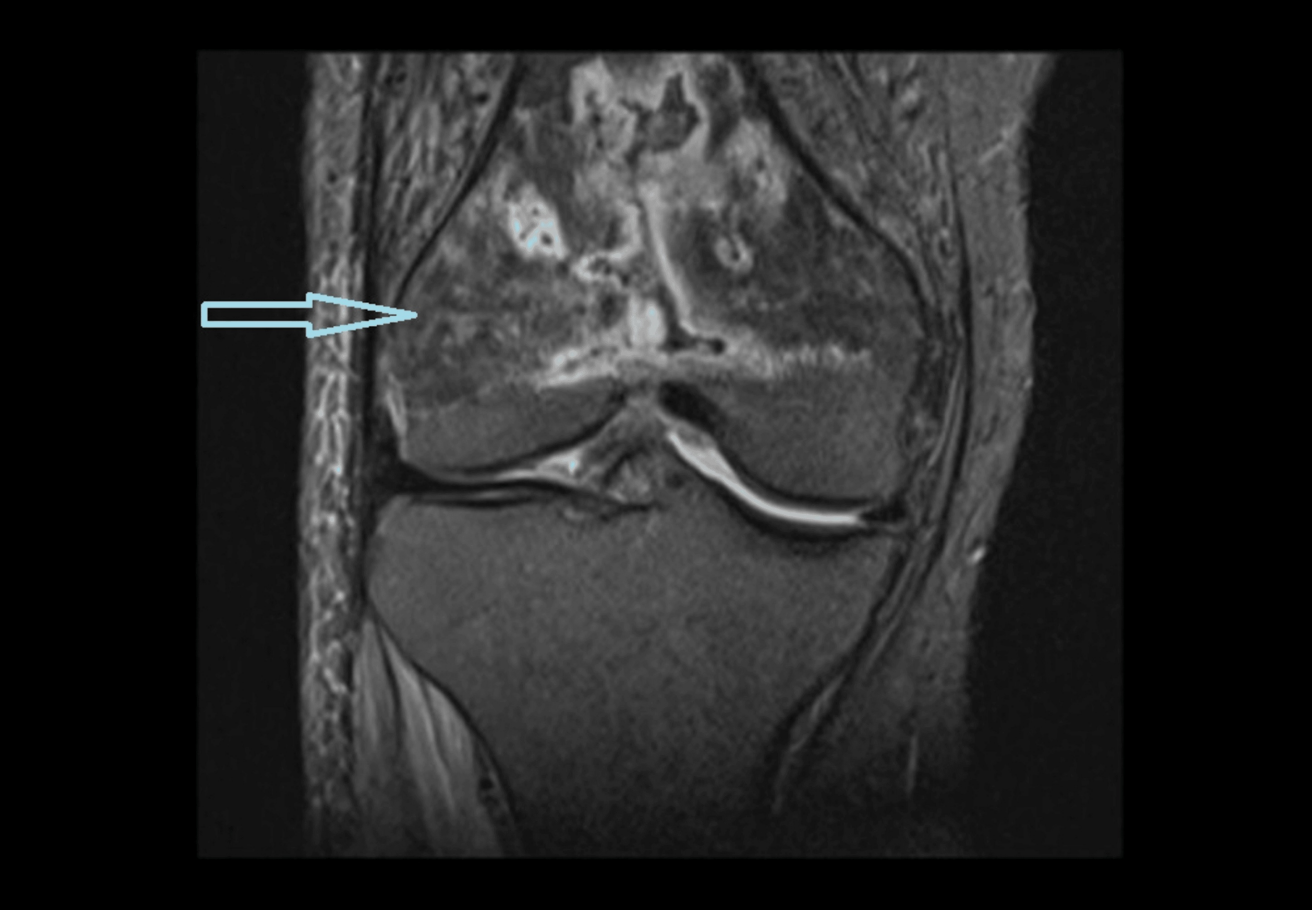
Proton density fat-saturated MRI sequence of the right knee showing features of osteomyelitis, with intramedullary collection and a cloaca involving the distal metaphysis and diaphysis of the right femur (arrow) MRI: magnetic resonance imaging.

To further characterize the bony lesion and post-debridement knee status, an MRI of the knee was performed, which revealed radiological features suggestive of chronic osteomyelitis of the right distal femur with no remnant abscess, as shown in Figure 3. An orthopedic consultation was obtained, and it was advised to continue medical management with antibiotics, with no immediate indication for surgical intervention.

**Figure 4:**
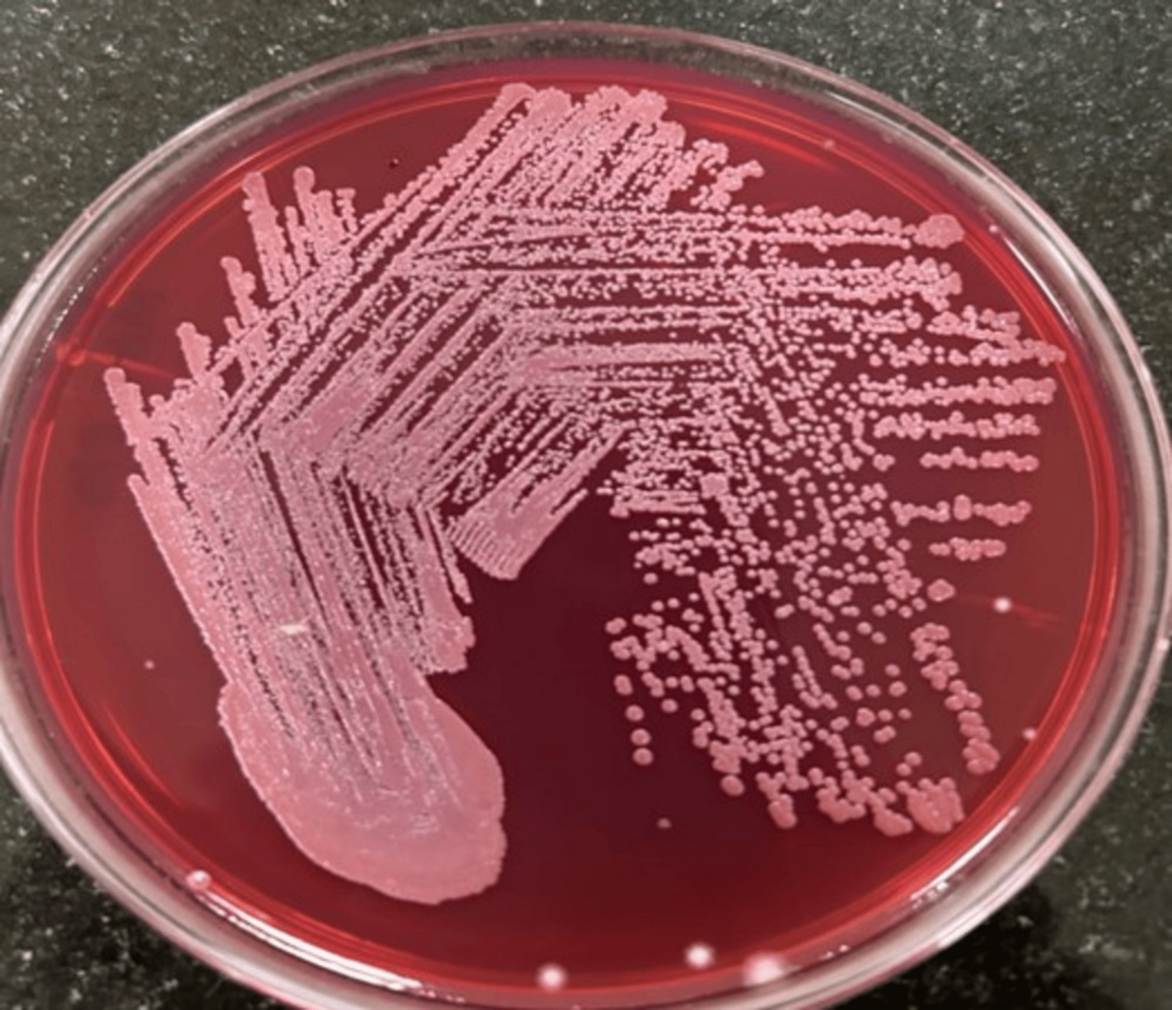
MacConkey agar showing growth of Burkholderia pseudomallei

On day 4 of admission, blood culture showed gray, dry, wrinkled colonies with hazy hemolysis on blood agar, and MacConkey agar revealed pink, dry, wrinkled colonies (Figure 4), suggestive of* B. pseudomallei.*

Species identification was performed using matrix-assisted laser desorption/ionization-time of flight (MALDI-TOF) mass spectrometry. Antibiotic susceptibility testing was conducted using the VITEK method, and the results were interpreted per CLSI M 100 guidelines. The isolate was sensitive to meropenem, co-trimoxazole, and ceftazidime.

As intensive therapy, injection meropenem 6 g/day was continued for four weeks, and as eradication therapy, tablet co-trimoxazole (160/800 mg) was added. Blood sugars were managed with insulin therapy. Following treatment, the frequency and intensity of febrile episodes gradually decreased, and by day 13 of admission, the patient became afebrile with complete improvement in sensorium. He was discharged in stable condition with a prescription for oral co-trimoxazole (160/800 mg) for six months. The patient was advised to have a diabetic diet, and insulin was continued for diabetes management. 

At one-month follow-up, the patient remained clinically stable. His pain was minimal (score 1/10). He resumed all his daily activities and returned to his job.

**Figure 5:**
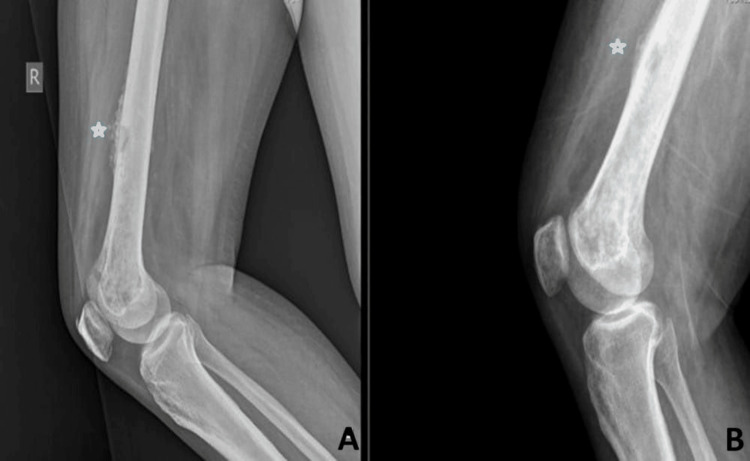
X-ray of the right femur (lateral view) showing osteomyelitis with periosteal thickening before antibiotics usage (A, asterisk) and resolution in osteomyelitis with minimal periosteal reaction after treating with antibiotics for one month (B, asterisk)

Repeat blood cultures done after intensive and eradication therapies were sterile. A repeat X-ray of the right lower limb revealed significant resolution in osteomyelitis of the right femur, as shown in Figure 5.

## Discussion

Literature review

A literature search was conducted to identify case reports of osteomyelitis caused by *B. pseudomallei*. All cases reported between 1995 and 2024 were screened, and twenty-one relevant articles were included in this brief review (Table 2).

**Table 2:** List of case reports of melioidosis with osteomyelitis ^*^Antibiotics were given as per culture sensitivity, and sensitive drugs were documented. ^**^Antibiotics were given as per culture sensitivity, and sensitive drugs were not documented. ^#^Antibiotics were given, but correlation with culture sensitivity was not specified.

Article		Age (years)/sex		Duration of symptoms	Immune status		Joint/bone involved		Antibiotics and duration	
Panigrahi et al. (2024) [14]		42/M		10 days	Details not mentioned		Right femur osteomyelitis		Intravenous meropenem and doxycycline^#^	
Mabayoje et al. (2022) [15]		33/M		2 months	Diabetes mellitus		Left femur shaft; metaphysis osteomyelitis		Ceftazidime for two weeks; co-trimoxazole for six months^*^	
Jayakumar and P (2022) [16]		47/M		2 weeks	Diabetes mellitus		Left hip joint		Ceftazidime 2 g for three weeks and oral co-trimoxazole for six months^#^	
Fairhead et al. (2020) [17]		61/M		4 days	Rheumatoid arthritis on methotrexate; cutaneous lupus erythematosus		L3/L4 discitis and vertebral osteomyelitis (right lung and lingula pneumonia)		Injection meropenem, followed by ceftazidime and oral trimethoprim-sulfamethoxazole for six weeks, followed by oral trimethoprim-sulfamethoxazole for six months^*^	
Prasad et al. (2020) [18]		38/M		2 months	Not immunocompromised		Right proximal tibia (liver, spleen abscess)		Ceftazidime 2 g, along with levofloxacin 750 mg for six weeks; co-trimoxazole double-strength tablet and doxycycline 100 mg for six months^**^	
Saluja et al. (2019) [19]		45/M		3 months	Diabetes mellitus		Bilateral knee, right ankle, left elbow osteomyelitis		Ceftazidime for four weeks; co-trimoxazole for six months^**^	
Alexander et al. (2018) [20]		45/M		9 months	Diabetes mellitus		Bilateral osteomyelitis of the distal femur, tibia, and fibula		Ceftazidime for six weeks; trimethoprim-sulfamethoxazole for six months^#^	
Huang et al. (2018) [21]		38/M		4 months	Not immunocompromised		Right femur osteomyelitis		Ceftazidime for four weeks; co-trimoxazole for six months^#^	
Conrad et al. (2016) [22]		25/M		3 weeks	Not immunocompromised		Right knee and right wrist		Intravenous ceftazidime and oral co-trimoxazole; ceftazidime was switched to oral levofloxacin on the fifth month; levofloxacin-co-trimoxazole was switched to doxycycline on the 10th month^#^	
Shetty et al. (2015) [23]		55/M		15 days	Uncontrolled diabetes mellitus		Right parietal bone		Ceftazidime 2 g; meropenem 2 g; oral trimethoprim-sulfamethoxazole 8/40 mg/kg twice daily after two weeks^#^	
James et al. (2013) [24]		9 months		45 days	Not immunocompromised		Fifth metatarsal		Ceftazidime for two weeks^#^	
Jane et al. (2012) [25]		52/M		5 days	Diabetes mellitus		Right femur (splenic abscess, lung fibrosis)		Meropenem with oral co-trimoxazole for 10 weeks, followed by a 12-month course of oral co-trimoxazole^#^	
Redondo et al. (2011) [26]		50/M		5 years	Diabetes mellitus		Right parietal bone (right knee septic arthritis)		IV cefepime and IV trimethoprim-sulfamethoxazole; oral doxycycline (100 mg every 12 hours); trimethoprim-sulfamethoxazole (one double-strength tablet every 12 hours) was given for one year^*^	
Bommakanti et al. (2010) [27]		52/M		Not mentioned	Details not mentioned		Calvarium of the skull		Ceftazidime (40 mg/kg) for two weeks, followed by oral co-trimoxazole (8/40 mg/kg) for six months^*^	
Cahn et al. (2009) [28]		32/M		3 weeks	Diabetes mellitus		Right medial malleolus, calcaneus, and first metatarsus		Ceftazidime (2 g intravenously 4×/d) and trimethoprim-sulfamethoxazole (1,920 mg orally 2×/d) for four weeks, followed by trimethoprim-sulfamethoxazole (1,920 mg orally 2×/d) and doxycycline (100 mg orally 2×/d) for an additional 20 weeks^#^	
Kronmann et al. (2009) [29]		20/M		4 weeks	Not immunocompromised		Skull, wrist, left distal femur (spleen, liver, kidney abscess, pulmonary nodules)		Ceftazidime, trimethoprim-sulfamethoxazole, and doxycycline for 14 weeks, followed by doxycycline and trimethoprim-sulfamethoxazole for 12 months^#^	
Falade et al. (2008) [30]		64/M		4 months	Diabetes mellitus		Thoracic spine, paraspinal abscess		Meropenem for two weeks; oral co-trimoxazole^*^	
Miksić et al. (2007) [31]		40/M		6 weeks	Details not mentioned		Right parietal bone osteomyelitis		IV ceftazidime and oral trimethoprim-sulfamethoxazole for eight weeks; trimethoprim-sulfamethoxazole and doxycycline for four months^*^	
Mukhopadhyay et al. (2007) [32]		29/M		1 year	Not immunocompromised		Left femur		Cefotaxime for two weeks followed by oral cefixime for four weeks; oral co-trimoxazole 160 mg and trimethoprim 800 mg for eight weeks^*^	
Ng et al. (2006) [33]		32/M		6 months	Diabetes mellitus		Left distal femur (liver and splenic abscess)		Gentamicin-impregnated polymethylmethacrylate (PMMA); ceftazidime 2 g; amoxicillin-clavulanate 625 mg twice daily for six weeks; calcium hydroxyapatite blocks filled with ceftazidime powder 5 g*	
Kareem et al. (1995) [34]		38/M		20 months	Diabetes mellitus		Left tibia		Ceftazidime 1 g for two weeks^#^	

*B. pseudomallei* is a Gram-negative environmental bacterium and the causative agent of melioidosis, a life-threatening infection that is estimated to account for ∼89,000 deaths per year worldwide. Risk factors for melioidosis include diabetes mellitus, alcoholism, chronic lung and renal diseases, congestive heart failure, malignancy, and immunosuppressive therapy. Diabetes mellitus is a major risk factor for *B. pseudomallei *infection, affecting 23%-60% of melioidosis cases. It weakens the immune system by reducing macrophage bacterial-killing ability, suppressing CD4+ regulatory T cell production, and disrupting inflammatory signaling pathways, thereby increasing susceptibility to infection [9,12].

Melioidosis presents with diverse clinical manifestations. These presentations depend on the route of entry, bacterial load, and immune status of the patient. The most common presentations include pneumonia and pleuritis (40%-60%), followed by mycotic aneurysm (40%-60%). Abscess formation frequently occurs in various organs, including the neck (0%-30%), kidneys (14%-28%), spleen (10%-33%), and liver (10%-33%). Additionally, skin ulcers and soft tissue abscesses are seen in 13%-24% of cases, osteomyelitis in 4%-14% (with the femur, tibia, and knee joint being the most frequently affected sites), and brain abscess or encephalomyelitis in 1%-5% of patients. Parotid abscesses occur in 0%-30% of cases, particularly in pediatric patients [9]. Splenomegaly is a notable clinical manifestation in chronic melioidosis, often reflecting the prolonged course of infection and underlying immune response [35].

As per a retrospective study, hyponatremia occurs in 84.1% (severe hyponatremia in 17.4% of patients) of hospitalized patients with melioidosis. A higher incidence of hyponatremia was noted in older ages and patients with acute kidney injury (AKI). Severe hyponatremia was found to be an independent predictor of in-hospital mortality, need for mechanical ventilation, and prolonged ICU stay [36].

The diagnosis of melioidosis relies on clinical suspicion, epidemiological concurrence, and bacterial culture positivity. Growth of *B. pseudomallei* from the culture of any site is diagnostic of melioidosis [9,37]. 

*B. pseudomallei* are intrinsically resistant to penicillin, ampicillin, first- and second-generation cephalosporins, gentamicin, tobramycin, and streptomycin. As per the data, primary resistance to meropenem, ceftazidime, and co-trimoxazole is exceedingly rare. Intravenous antibiotic options include ceftazidime administered every 6-8 hours (or via 24-hour infusions) or meropenem every eight hours, particularly for critically ill patients or those with central nervous system involvement. The eradication phase, essential to prevent relapse, typically lasts for three to more than six months, with trimethoprim-sulfamethoxazole (TMP-SMX) as the first-line agent, or co-amoxiclav or doxycycline as alternatives. When melioidosis presents as osteomyelitis, longer treatment durations are recommended to reduce the risk of recurrence. Management involves an intensive phase of intravenous antibiotics, typically meropenem or ceftazidime, for at least 4-6 weeks, followed by an oral eradication phase using co-trimoxazole (with doxycycline as an alternative) for a minimum of six months [38,39].

## Conclusions

This case highlights the clinical presentation and management of a patient with chronic osteomyelitis, a rarer manifestation of melioidosis. Our patient, a poorly controlled diabetic and reformed alcoholic, presented with chronic osteomyelitis caused by *B. pseudomallei*. He had a notable history of exposure to contaminated water. Effective management of risk factors (such as diabetes), correction of hyponatremia, and guidance from positive cultures and sensitivity testing allowed for the selection of appropriate antibiotics. The patient was treated with an intensive phase of meropenem and TMP-SMX, followed by eradication therapy with TMP-SMX, leading to clinical recovery. This case highlights the critical role of early diagnosis, timely treatment, and targeted antimicrobial therapy in the successful management of melioidosis.
